# Higher Positive Affect is Associated with Lower Systemic Inflammation in Women but not in Men: Findings from MIDUS

**DOI:** 10.1093/geroni/igaf122.3954

**Published:** 2025-12-31

**Authors:** Rui Wang, Derek Spangler, Christopher Engeland, Harold Lee

**Affiliations:** The Pennsylvania State University, University Park, Pennsylvania, United States; The Pennsylvania State University, University Park, Pennsylvania, United States; The Pennsylvania State University, University Park, Pennsylvania, United States; The Pennsylvania State University, University Park, Pennsylvania, United States

## Abstract

Higher positive affect is associated with healthy aging, with inflammation proposed as a mediating mechanism. However, most studies assessed inflammation primarily via CRP and/or IL-6, which may insufficiently capture the breadth of systemic inflammation. We examined the association between positive affect and a cytokine composite consisting of four inflammatory cytokines. We hypothesized that higher positive affect would be associated with lower cytokine composite levels, and this association would vary based on sex given women’s higher inflammation. We used data from Midlife in the United States (N = 1023, mean age 46 yrs, female 54%). Positive affect was measured by Positive Affect and Negative Affect Schedule (range: 1-5, mean score: 3.67) between 2004 and 2006. The cytokine composite was assessed using IL-6, IL-8, IL-10, and TNF-α, and was collected between 2004 and 2009. The cytokine composite was regressed on positive affect, with covariates: time difference between positive affect and blood draw for cytokines, sociodemographic factors (e.g., age, sex), negative affect, and health behaviors and conditions (e.g., smoking, BMI). Positive affect was not associated with the cytokine composite in the total sample (r2=.14, b=-0.04, p = 0.06, 95% CI [-0.08, 0.01]). However, sex moderated the association (p = 0.03): among women, higher positive affect was associated with lower cytokine composite (r2=.13, b=-0.07, p = 0.02, 95% CI [-0.13, -0.01]), but no association was observed in men (r2=.15, b=-0.01, p = 0.70, 95% CI [-0.08, 0.05]). In women, higher positive affect was prospectively associated with lower systemic inflammation, which leads to healthy aging. Future studies should investigate mechanisms underlying this sex-specific association.

